# Effect of Lipopolysaccharide and Muramyl Dipeptide on Apoptosis of Bovine Mammary Gland Lymphocytes

**DOI:** 10.3390/ani10060990

**Published:** 2020-06-05

**Authors:** Petr Slama, Eliska Kabourkova, Zbysek Sladek, Terezie Zavadilova, Lucie Kratochvilova, Kristina Kharkevich, Shubhadeep Roychoudhury, Ales Pavlik, Andrea Roztocilova, Michal Uhrincat, Vladimir Tancin, Kazuhiro Kimura, Roman Konecny, Yoshio Kiku, Atsushi Watanabe, Jong-Young Kwak, Monika Zouharova

**Affiliations:** 1 Department of Animal Morphology, Physiology and Genetics, Faculty of AgriSciences, Mendel University in Brno, Zemedelska 1, 613 00 Brno, Czech Republic; zbysek.sladek@mendelu.cz (Z.S.); terezie.zavadilova@mendelu.cz (T.Z.); xkratoc3@node.mendelu.cz (L.K.); kristina.kharkevich@mendelu.cz (K.K.); shubhadeep1@gmail.com (S.R.); ales.pavlik@mendelu.cz (A.P.); 2 Department of Animal Origin Food and Gastronomic Sciences, Faculty of Veterinary Hygiene and Ecology, University of Veterinary and Pharmaceutical Sciences Brno, Palackeho tr. 1, 612 42 Brno, Czech Republic; KABOURKOVAE@vfu.cz; 3 Department of Life Science and Bioinformatics, Assam University, Silchar 788 011, India; 4 Department of Animal Nutrition and Forage Production, Faculty of AgriSciences, Mendel University in Brno, Zemedelska 1, 613 00 Brno, Czech Republic; andrea.roztocilova@mendelu.cz; 5 NPPC-Research Institute for Animal Production, Hlohovecka 2, 951 41 Luzianky, Slovakia; michal.uhrincat@nppc.sk (M.U.); vladimir.tancin@uniag.sk (V.T.); 6 Department of Veterinary Sciences, Faculty of Agrobiology and Food Resources, Slovak University of Agriculture in Nitra, Trieda A. Hlinku 2, 949 76 Nitra, Slovakia; 7 Laboratory of Biochemistry, Faculty of Veterinary Medicine, Hokkaido University, Sapporo, Hokkaido 060-0818, Japan; k-kimura@vetmed.hokudai.ac.jp; 8 Department of Animal Husbandry Sciences, Faculty of Agriculture, University of South Bohemia in Ceske Budejovice, Studentska 1668, 37005 Ceske Budejovice, Czech Republic; konecnyroman@centrum.cz; 9 Hokkaido Research Station, National Institute of Animal Health, National Agriculture and Food Research Organization, 4 Hitsujigaoka, Toyohira, Sapporo, Hokkaido 062-0045, Japan; yokiku@affrc.go.jp (Y.K.); awata@affrc.go.jp (A.W.); 10 Department of Pharmacology, School of Medicine, Ajou University, Suwon 16499, Korea; jykwak@ajou.ac.kr; 11 Department of Immunology, Veterinary Research Institute, Hudcova 70, 621 00 Brno, Czech Republic; zouharova.m@vri.cz

**Keywords:** lipopolysaccharide, *Escherichia coli*, muramyl dipeptide, mastitis, apoptosis, lymphocyte, mammary gland, CD44

## Abstract

**Simple Summary:**

Inflammation of the mammary gland in dairy cattle is a global problem and causes huge financial loss to dairy farmers. Inflammation is caused by many species of bacteria penetrating through the teat canals into the udder. Those bacteria are usually eliminated by treatment with intramammary injection of antibiotics, while they are also eliminated by the immune cells of the cow. One of the immune cells are lymphocytes which are responsible for specific immunity. When viable, they are able to carry out their normal functions. The present study focused on the investigation of cell death of lymphocytes during bovine mammary gland inflammation. We analyzed apoptosis in mammary gland lymphocytes under the stimulation of lipopolysaccharides and muramyl dipeptide as the endotoxin of Gram-negative bacteria and the natural content of the cell wall of Gram-positive bacteria. We found that they induce lymphocyte apoptosis in the early phase of inflammation, which can be associated with the expression of CD44 receptors on lymphocytes. This receptor is important in many physiological processes, including apoptosis of cells. For a better understanding of immune responses in mammary glands and for developing of immunotherapy without antibiotics, the process of inflammation, including cell death of immune cells necessitates further holistic studies.

**Abstract:**

The aim of this study was to evaluate whether apoptosis of lymphocytes is modulated by stimulation by lipopolysaccharide (LPS) of *Escherichia coli* or muramyl dipeptide (MDP). Cell populations were obtained by lavaging of the mammary glands 24, 48, 72, and 168 h following intramammary induced inflammation. The portion of apoptotic lymphocytes peaked at 48 h after treatment with LPS or MDP. The analysis of CD44 expression of the same cell populations showed a higher percentage of CD44-positive lymphocytes 24- and 48-h following induction of inflammation by LPS or MDP. The results demonstrate that during both experimental infection of bovine mammary glands with LPS or MDP, apoptosis of lymphocytes was induced in the initial phase of the inflammatory response and CD44 was also overexpressed at the beginning of inflammation. These data suggest a connection of lymphocyte apoptosis with the expression of CD44 receptors.

## 1. Introduction

Bacteria are the most important pathogens which are able to cause inflammation of the mammary gland (mastitis). In cattle, there are many bacterial pathogens causing mastitis and, therefore, a high negative impact on dairy farming [1,2,3]. The bacterial pathogens are distinguished into two groups, Gram-positive and Gram-negative bacteria. Such bacteria have pathogen-associated molecular patterns (PAMPs), which are recognized by immune cells using pattern recognition receptors (PRRs). Endotoxin lipopolysaccharide (LPS) is a typical PAMP of Gram-negative bacterium as *Escherichia coli*. The typical PAMP of Gram-positive bacteria is peptidoglycan. Muramyl dipeptide (MDP) is the minimal structural unit of peptidoglycans [4]. One of the major Gram-negative pathogens causing bovine mastitis is *E. coli* [5]. In the group of major Gram-positive bacteria, which are able to cause bovine mastitis, there are two important pathogens causing a lot of inflammation of mammary glands: *Staphylococcus aureus* and *Streptococcus uberis* [6]. Their pathogenicity is given by their special mechanisms to avoid direct elimination by immune cells. *S. aureus* uses the ability to survive inside the phagosomes of neutrophils and through a mechanism to avoid the fusion of phagosomes and lysosomes in neutrophils. When neutrophils die by necrosis, their contents spread out, which is harmful to the surrounding tissues and may further bring about a second wave of attack [7]. *Str. uberis* very “cleverly” use the *Streptococcus uberis* adhesion molecule (SUAM) to internalize into epithelial cells [8,9,10,11,12]. These bacteria are hidden inside epithelial cells without causing any other physiological problem. These bacteria are not affected by antibiotics under these conditions and can survive inside the epithelial cells until the epithelial cells die. Then, these bacteria spread out and cause further damage to tissues. Such a mechanism allows them to partially overcome the non-specific immune response. Subsequently, the specific immune response has to play a role of eradication of these antigens. A specific immune response is elicited by lymphocytes. T and B cells have different mechanisms for eliminating antigens [4]. It is obvious those immunocompetent cells must be viable to perform their functions. Various bacteria are able to modulate the apoptosis of lymphocytes in different tissues [13,14]. *S. aureus* and *Str. uberis* can delay apoptosis of lymphocytes in the mammary gland. A gradual increase of apoptosis in lymphocytes during inflammation of the bovine mammary gland has been reported [15]. In another study, we investigated the effect of peptidoglycan of *S. aureus* on apoptosis of lymphocytes and the apoptosis of lymphocytes was found to be induced in the initial stage of mastitis [16]. The distribution of different subpopulations of lymphocytes in mammary gland secretions also changes during lactation or inflammation [17,18]. In another previous study, Sustrova and Slama [19] detected an increase of gamma delta T cells during an inflammatory response caused by *S. aureus*. The increase of apoptotic lymphocytes also correlated with the rise in the portion of gamma delta T cells in the inflammatory secretions [20]. Furthermore, *S. aureus* is capable of producing enterotoxins that are harmful to mammary gland tissue and contribute to the development of inflammation [21]. *S. aureus* alpha-toxin is known as an inducer of apoptosis in human peripheral blood mononuclear cells [22], and staphylococcal enterotoxin C is also able to affect apoptosis of bovine lymphocytes [23].

CD44 is the cell-surface glycoprotein receptor for hyaluronan [24]. It has multiple functions in organisms, including lymphocyte activation and apoptosis [25,26,27]. CD44 is also involved in the pathogenesis of bacterial infections, including streptococcal infections [25,28]. Functions of CD44 during inflammation of the bovine mammary gland in connection with neutrophils and macrophages have been investigated previously [29,30,31]. However, detection of CD44 on lymphocytes during mastitis and the role of this cell-surface glycoprotein on the course of lymphocyte apoptosis in the inflammatory response of mammary gland has not yet been studied in great detail.

The goal of the present study was to evaluate the effect of LPS and MDP on the apoptosis and CD44 expression in lymphocytes during the inflammatory response of the bovine mammary gland.

## 2. Materials and Methods 

### 2.1. Animals

For our experiments, we used 10 virgin heifers (crossbred Holstein × Bohemian Red Pied) of 16 to 18 months old—five heifers with each of the inducers LPS and MDP, respectively. All experiments with animals were approved by the Branch Commission for Animal Welfare of the Ministry of Education, Youth and Sports of the Czech Republic (MSMT-11516/2019-2). Experimental animals were free of intramammary infections, which was demonstrated by a bacteriological examination of mammary gland lavages. The bacteriological examination of mammary gland lavages was executed through culture on blood agar plates with aerobic incubation at 37 °C for 24 h.

### 2.2. Experimental Design

For a control for infections, there were used phosphate-buffered saline (PBS; Sigma, Saint Louis, Missouri, USA) with a volume of 20 mL for each mammary gland [15,29]. All mammary gland sinuses of each udder were washed out with PBS to obtain a cell suspension using the following procedure. The first sample was obtained by lavage of the left forequarter 24 h following instillation of PBS. The remaining quarters were washed out at 48 h (left-rear), right-front at 72 h, right-rear at 168 h following the use of PBS. The differential cell count of leukocytes obtained from the lavages was analyzed by flow cytometry using forward scatter and side scatter in dot plots [32]. The total cell count was detected using the Fossomatic 90 analyzer (Foss Electric, Hillerod, Denmark).

### 2.3. Induction of Inflammatory Response

In our experiments, we used LPS of *Escherichia coli* (serotype 0128:B12; Sigma, Saint Louis, Missouri, USA) at a concentration of 5 µg in 20 mL PBS and MDP (MurNAc-L-Abu-D-IsoGln, Institute of Organic Chemistry and Biochemistry of the Czech Academy of Sciences, Prague, Czech Republic) at a concentration of 500 µg in 20 mL PBS. The external part of teat canal of all mammary glands were cleansed with 70% ethanol, and the mammary glands were washed out using catheters (AC5306CH06, Porges S.A., Le Plessis Robinson, France) in the same manner as PBS treating.

### 2.4. Flow Cytometry Analysis of Apoptosis and CD44

Apoptotic lymphocytes were analyzed by flow cytometry (FACSCalibur apparatus, Becton Dickinson, San Jose, CA, USA) following staining with Annexin-V (FITC) and propidium iodide (PI) [33]. For that purpose, we used the Annexin-V-FLUOS staining kit (Boehringer Mannheim, GmbH, Mannheim, Germany). Five hundred µL of the incubation buffer was mixed with 10 µL of FITC-Annexin-V and 10 µL of PI solution. After 15 min of incubation at room temperature, the cell suspension was analyzed by flow cytometry with differentiation of 20,000 cells. Lymphocytes were distributed over three different quadrants of dot plots representing viable (Annexin-V-/PI-), apoptotic (Annexin-V+/PI-), and necrotic lymphocytes (Annexin-V+/PI+). The percentages of apoptotic lymphocytes were calculated from the total number of lymphocytes. The dot plots were assessed using the WinMDI software (Windows Multiple Document Interface for Flow Cytometry; Purdue University, West Lafayette, IN, USA).

For analysis of CD44, mouse anti-ovine antibody CD44 BAG40A (VMRD Inc. Pullman, Washington, USA) diluted 1:50 and FITC labelled IgG3 (SouthernBiotech, Birmingham, Alabama, USA) diluted 1:100 as the primary and the secondary antibodies were used, respectively.

### 2.5. Statistical Analysis

Arithmetic means and standard deviations were used to describe the total cell count, differential cell count, apoptotic lymphocytes and CD44 positive lymphocytes. Statistically significant differences in the portions of the mentioned parameters were assessed using the paired t-test. The relationship between apoptosis of lymphocytes, and CD44 expression was ascertained by correlation analysis (Pearson correlation coefficient). The data were analyzed by the STATISTICA 8.0 software (StatSoft CR Ltd., Prague, Czech Republic).

## 3. Results

### 3.1. The inflammatory Response of the Mammary Gland to LPS and MDP

The inflammatory response was induced by LPS or MDP. We analyzed cell populations obtained by lavages at four time points following induction by the above-mentioned agents (Table 1). We noticed that LPS elicited an influx of leukocytes, especially neutrophils (Table 2), more intensively than MDP. The total cell count was elevated significantly using both inducers of inflammation, LPS (*P* < 0.01), or MDP (*P* < 0.05), in comparison with the control at 24 h following infection. After 48 and 72 h, a significant increase was noted only for LPS (*P* < 0.05) (Table 1).

The proportion of lymphocytes in the differential cell count was changed significantly after LPS induction of mammary glands after 24, 48, and 168 h, respectively (*P* < 0.01). The changes in the proportion of lymphocytes in the MDP induction were not significant in comparison with the control (Table 2).

### 3.2. The Dynamics of Lymphocyte Apoptosis and CD44 Expression on Lymphocytes During LPS and MDP Infection

**Figure 1:**
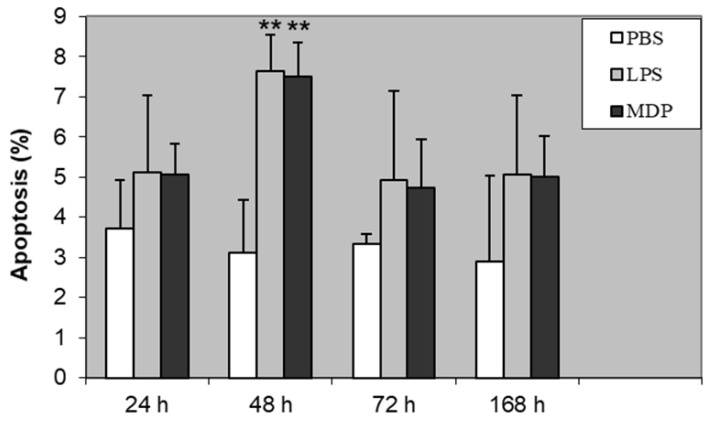
The proportion of apoptotic lymphocytes following induction of bovine mammary glands with PBS (control), LPS, and MDP. Statistically significant differences between control (PBS) and LPS or MDP is marked with asterisks (** *P* < 0.01).

We analyzed apoptosis of lymphocytes and expression of CD44 receptor in 24, 48, 72, and 168 h following intramammary induction with PBS (control), LPS, and MDP. Apoptosis of lymphocytes significantly increased 48 h after experimental infection, both in LPS and MDP groups, contrary to the control (*P* < 0.01). At all other time points, an increasing trend in lymphocyte apoptosis was noted, although without any statistically significant difference (Figure 1).

**Figure 2:**
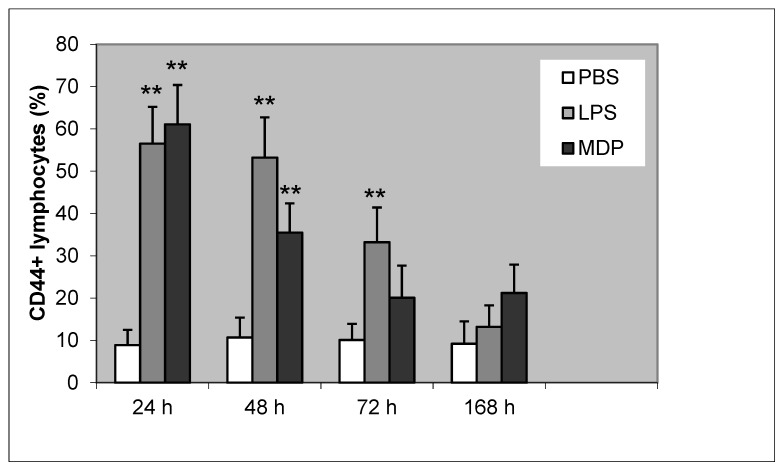
Proportion of CD44+ lymphocytes following induction of bovine mammary gland with PBS (control), LPS, and MDP. Statistically significant differences between the control (PBS) and LPS or MDP is marked with asterisks (** *P* < 0.01).

Elevation of CD44 positive lymphocytes was noted after 24, 48, 72, and 168 h of mammary gland induction of inflammation with LPS or MDP. After 24 and 48 h, the increase in CD44 expression was statistically significant for both LPS and MDP (*P* < 0.01). After 72 h, the increase was significant only for LPS (*P* < 0.01). At the last time point (168 h), the increase in CD44 expression was not statistically significant (Figure 2). We noted a high correlation between lymphocyte apoptosis and CD44 positive lymphocytes after induction using LPS (r^2^ = 0.912; *P* < 0.01) and MDP (r^2^ = 0.902; *P* < 0.01), respectively.

## 4. Discussion

In this study, we analyzed the possible effect of LPS and MDP on the apoptosis of lymphocytes and the expression of cell membrane receptor CD44 on lymphocytes during the experimentally induced inflammatory response of bovine mammary gland. We also detected the total cell counts and differential cell counts of mammary lavages following intramammary instillation of LPS and MDP. In our experiments, we used 5 μg of LPS or 500μg of MDP. Such concentrations have been chosen because they have been tested and used before [30,34]. We used lower concentrations of LPS than MDP because it is a stronger inducer of inflammation.

We noted a rapid increase of total cell count in inflammatory response caused by LPS or MDP in comparison with control samples. The biggest increase occurred after using LPS. These findings are not surprising, and they are consistent with previous studies [30,34]. The lymphocyte ratio in the differential cell count gradually increased after LPS induction in comparison with controls; and also after MDP induction, where the increase in lymphocyte ratio was greater. It is known that the lymphocyte ratio changes during lactation [17] and inflammation [15,18].

Stimulation of the mammary gland by LPS or MDP caused statistically significant enhancement of the proportion of apoptotic lymphocytes 48 h following the induction of the inflammatory response. A similar trend was reported in a previous study on the effect of peptidoglycan on lymphocyte apoptosis [16]. However, a gradual increase in lymphocyte apoptosis was noted while using *S. aureus* or *Str. uberis* for the induction of inflammation [15]. The effect of living bacteria does not seem to be as pronounced as using the components of their cell wall or endotoxins for induction of lymphocyte apoptosis during experimentally induced inflammation of the mammary gland.

The expression of CD44 on lymphocytes was rapidly elevated during the inflammatory response compared with controls. The highest percentages of CD44 positive lymphocytes were shown 24- and 48-h following induction of the mammary gland by both LPS and MDP. Similar results were reported while using peptidoglycan, too [35]. In our study, a statistically significant positive correlation was noted between lymphocyte apoptosis and CD44 expression of lymphocytes 48 h following induction of inflammation by both LPS and MDP. These findings seem to indicate a relationship between the CD44 receptor and induction of apoptosis. Although such interpretation is not unanimous given some previous reports of involvement of CD44 in anti-apoptotic mechanisms [25,36,37]. CD44 inhibits apoptosis by sequestering CD95, putting an end to the development of the death-inducing signaling complex [38]. However, Pilon-Thomas et al. [36], Rajasagi et al. [37], and Jordan et al. [25] referred to the functions of CD44 in connection with chronic diseases and cancer. CD44 seems to be involved in the survival signaling of chronic lymphocytic leukemia, too [39,40,41,42,43]. Despite these facts, the results of the present study led us to hypothesize that CD44 could be involved in the induction of lymphocyte apoptosis in acute inflammation. Furthermore, Jordan et al. [25] reported multiple functions of CD44 in various diseases. A number of in vitro studies revealed the functions of CD44 in the induction or prevention of cell death [38,44,45,46]. Moreover, there are multiple isoforms of CD44 with possibly different functions [25,27,47]. Foger et al. [48] reported that CD44 supports T cell proliferation and apoptosis by apposition of protein kinases. The authors further postulated that CD44 supports T cell apoptosis not only by clustering of the TCR/CD3 complex and provisioning of Src family kinases but also by recruiting additional membrane receptors. CD44 may be involved in aberrant signaling pathways leading to apoptosis not only in lymphocytes but also in other cells, for example, in β cells in type 1 diabetes [49].

CD44 expression is regulated by the family of mitogen-activated protein kinases, which play a crucial role in proliferation, differentiation, and apoptosis of cells [50,51]. CD44 expression is up-regulated in monocytes by TNF-α, and this cytokine is produced following induction by LPS [51]. Assuming the existence of a similar mechanism in lymphocytes, LPS intramammary infusion can elicit the influx of neutrophils, which is the principal source of TNF-α production [52,53], and this cytokine then can up-regulate the expression of CD44 in lymphocytes. Gee et al. [51] pointed out that the understanding of the mechanism behind CD44 induction may be helpful in the development of strategies for the modulation of inflammatory responses. Other pro-inflammatory cytokines, such as IL-1 and IL-6 are also capable of inducing lymphocyte apoptosis [54]. However, IL-7 elicits an opposite response by inhibiting the apoptosis of lymphocytes [55]. Therefore, it is necessary to study the cytokine network of inflammatory diseases in great detail to exactly understand the mechanisms behind cell death and expression of receptors related to apoptosis of cells.

## Figures and Tables

**Table 1:** Total cell count (mean ± SD; 10^6^ per mL) during the inflammatory response following intramammary application of phosphate-buffered saline (PBS), lipopolysaccharide (LPS), and muramyl dipeptide (MDP).

Agent	24 h	48 h	72 h	168 h
PBS	12.1 ± 2.9	5.1 ± 1.9	3.2 ± 1.5	1.3 ± 0.7
LPS	45.3 ± 9.8 **	18.2 ± 9.0 *	7.2 ± 3.1 *	1.8 ± 0.6
MDP	31.5 ± 5.9 *	11.9 ± 5.8	4.5 ± 3.0	1.5 ± 0.5

Statistically significant differences between control (PBS) and LPS or MDP is marked with asterisks (** *P* < 0.01, * *P* < 0.05).

**Table 2:** Differential cell count (mean ± SD; %) during the inflammatory response following intramammary application of PBS, LPS, and MDP.

Agent	Cells	24 h	48 h	72 h	168 h
PBS	Lymphocytes	7.5 ± 4.2	18.7 ± 12.9	17.2 ± 10.5	9.3 ± 4.7
	Macrophages	30.1 ± 9.8	36.1 ± 7.3	55.3 ± 11.6	80.3 ± 12.0
	Neutrophils	62.4 ± 7.2	45.2 ± 13.2	27.5 ± 8.6	10.4 ± 4.2
LPS	Lymphocytes	2.1 ± 0.7 **	6.3 ± 2.7 **	16.2 ± 7.4	24.7 ± 10.6 **
	Macrophages	12.7 ± 3.1 **	13.2 ± 5.6 **	32.7 ± 13.3 *	50.4 ± 9.8 *
	Neutrophils	85.2 ± 8.8 **	80.5 ± 12.3 **	51.1 ± 14.4 **	24.9 ± 9.3 **
MDP	Lymphocytes	6.7 ± 3.8	18.9 ± 6.8	24.5 ± 8.0	21.4 ± 9.5
	Macrophages	11.2 ± 4.3 **	25.6 ± 7.6	50.2 ± 12.3	62.8 ± 12.9 *
	Neutrophils	82.1 ± 9.6 **	55.5 ± 10.7	25.3 ± 8.2	15.8 ± 6.2 *

Statistically significant differences between control (PBS) and LPS or MDP is marked with asterisks (** *P* < 0.01, * *P* < 0.05).
